# Differences between *Staphylococcus aureus* nasal carriage and IgE-sensitization to *Staphylococcus aureus* enterotoxin on risk factors and effects in adult population

**DOI:** 10.1186/s13223-022-00648-4

**Published:** 2022-01-31

**Authors:** Han-Ki Park, Seok-Ju Yoo, Taek Soo Kim, Byung-Keun Kim, Sekyung Jang, Sung Yeon Kim, Kwan Lee

**Affiliations:** 1 Department of Allergy and Clinical Immunology, School of Medicine, Kyungpook National University, Daegu, Republic of Korea; 2 Department of Preventive Medicine, College of Medicine, Dongguk University, 123 Dongdae-ro, Gyeongju, 38066 Republic of Korea; 3 Department of Laboratory Medicine, Seoul National University College of Medicine, Seoul, Republic of Korea; 4 Department of Internal Medicine, Korea University College of Medicine, Seoul, Republic of Korea; 5 Environmental Health Research Division, National Institute of Environmental Research, Seo-gu, Incheon, 22689 Republic of Korea

**Keywords:** *Staphylococcus aureus*, Staphylococcal enterotoxins, Risk factors, Immunoglobulin E, Eosinophil, Cough, Sputum

## Abstract

**Background:**

*Staphylococcus aureus* (SA) nasal carriage (SA carriage) and IgE-sensitization to SA enterotoxin (SE IgE-sensitization) are known to be associated with chronic airway disease.

**Objective:**

This study aimed to evaluate the differences in risk factors, type 2 inflammation and respiratory symptoms between SA carriage and SE IgE-sensitization.

**Methods:**

We conducted a cross-sectional study of a community-based adult population to evaluate the environmental exposure and health impact of the Pohang Industrial Complex, Korea. Participants were examined based on self-reported questionnaires, nasal swab, and blood sampling.

**Results:**

There were 307 participants, and the overall prevalence of SA carriage and SE IgE-sensitization was 26.1% (80/307) and 25.7% (79/307), respectively. An urban environment was significantly correlated with SA carriage, whereas age and obesity were significantly correlated with SE IgE-sensitization. SA carriage was not associated with an increase in total IgE and blood eosinophil count, whereas SE IgE-sensitization was associated with an increased total IgE and blood eosinophil count. SA carriage was significantly correlated with cough persisting for more than three weeks (OR, 3.044; 95% CI, 1.137–8.153) and sputum (OR, 2.429; 95% CI, 1.008–5.854). SE IgE-sensitization was a significant correlation with only sputum (OR, 2.452; 95% CI, 1.066–5.640). SA carriage and SE IgE-sensitization showed a synergistic effect on the prevalence of cough and sputum.

**Conclusion:**

SA carriage was associated with the urban environment, and SE IgE-sensitization was associated with the elderly and obesity. SA carriage and SE IgE-sensitization had different correlation with type 2 inflammation and airway symptoms.

**Supplementary Information:**

The online version contains supplementary material available at 10.1186/s13223-022-00648-4.

## Introduction

*Staphylococcus aureus* (SA) is one of the most colonized strains in the environment and human body surfaces and it can act as a facultative bacterial pathogen [1]. The nasal cavity is the most common colonization area of SA in the human body, and the permanent carriage in more than 20% of the general population [2]. SA in the nasal cavity interacts with another commensal microbiome, nasal epithelial cells, and other immune cells [1, 3]. Therefore, SA nasal carriage (SA carriage) is known not only to increase the risk of opportunistic infections, but also to increase the risk of chronic airway diseases.

Recently, SA has been suggested as a possible mechanism of type 2 inflammatory response with eosinophils. One of the mechanisms of SA that induces type 2 inflammation is the formation of IgE antibodies against SA superantigen (SA enterotoxin, SE). SE stimulates polyclonal IgE formation. SE IgE directly activates mast cells and basophils and stimulates the secretion of type 2 cytokines such as IL-4, IL-5, and IL-13 [4]. Indeed, SE IgE-sensitization is highly correlated with the chronic rhinosinusitis with nasal polyp and eosinophilic asthma, which are associated with airway type 2 inflammation [5]. However, unlike SE IgE-sensitization, the correlation with type 2 inflammation is not clear with SA carriage alone [6, 7], and it may appear as a correlation with other airway inflammations pattern (i.e., neutrophilic inflammation) [8].

We know that SA carriage and SE IgE-sensitization are biomarkers related to chronic airway inflammatory disease, but the difference between the two has not been well characterized so far. There are not enough studies on how the environmental risk factors of SA carriage and SE IgE differ. Furthermore, knowledge of the different effects on type 2 inflammation and airway symptoms of SA carriage and SE IgE-sensitization is lacking. Therefore, we conducted health assessments of individuals living in three areas near the Pohang Industrial Complex in Korea to confirm the difference between SA carriage and SE IgE-sensitization in adults. We identified and compared the association factors including environment, social, and lifestyle factors of SA carriage and SE IgE-sensitization and investigated correlations between SA carriage, SE IgE, and SE IgG. In addition, we examined whether type 2 inflammation markers and respiratory symptoms were increased in the SA carriage and SE IgE-sensitization groups.

## Methods

### Study participants

Voluntary participants in Pohang City, Korea, near the Pohang Industrial Complex were enrolled in this cross-sectional study and responded to a questionnaire to evaluate environmental exposure and health impacts. They were divided into three environmental groups based on considerations of distances of their residences from the Pohang Industrial Complex and the level of exposure to air pollutants including fine dust (Additional file 1: Figure S1A, B). These three groups were classified as an urban area group, a suburban area group, and a rural area group. In the urban area group, not only the total amount of fine dust but also the concentrations of heavy metals and volatile organic compounds (VOCs) as well as air pollutants such as SO_2_, NO_2_ and CO were higher. Furthermore, those in the urban area group lived in a high air pollution area with a population density of 100–10,000 (persons/km^2^), whereas the rural area group lived in a low air pollution area with a population density of ≤ 60 (persons/km^2^) (Additional file 1: Figure S1C). The registered participants were adults (≥ 19 years old) who had lived in their respective areas for ≥ 5 years. Those who worked for > 8 h/day in an area outside of the residence area and those with communication difficulties were excluded. The Institutional Review Board of Dongguk University Gyeongju Hospital reviewed and approved the study protocol before the survey (approval number: 19-093). The aims and objectives of the study were explained before the enrollment to all participants, and the written and informed consent were obtained from them.

### Data collection and definitions

Information on the subjects’ environment characteristics (e.g., home type, proximity to main roadways, etc.) and lifestyle characteristics (e.g., smoking, alcohol consumption, exercise habits, and diet, etc.) were collected using a questionnaire. Underlying comorbidities, including asthma and allergic rhinitis, were defined based on the answers given in the questionnaire: (e.g., “Have you ever been diagnosed with asthma by doctor?”).

Cough was defined as a cough that lasted more than 3 weeks to exclude acute upper respiratory infections. Respiratory symptoms were determined based on responses to questionnaire items as follows: cough (“Do you have an uncomfortable cough that lasts more than 3 weeks?”); sputum production (“Do you find sputum production uncomfortable?, Exclude caused by acute factors such as upper respiratory infection.”); dyspnea (“Have you experienced dyspnea over the last 12 months?”); wheezing (“Have you experienced wheezing at any point in the last 12 months?”).

Blood and nasal swab samples were obtained from all participants and were subjected to various analyses, such as complete blood count, blood urine nitrogen, creatinine, total IgE, SE immunoglobulin assays and SA culture.

### *Staphylococcus aureus* culture

Nasal swabs were obtained from the anterior nares using Amies agar gel transport swabs. Transported specimens were incubated using blood agar plates (Asan Pharmacy, Seoul, Korea) for 2 days at 35 °C. Gram-staining was performed on isolated colonies, and when Gram-positive cocci were confirmed, the matrix-assisted laser desorption/ionization time-of-flight (MALDI-TOF) analysis was performed using Biotyper (Bruker Daltonik GmbH, Bremen, Germany) to confirm the presence of SA. A positive result for the SA culture was defined as SA carriage.

### Measurements of SE-specific IgE and IgG

Measurements of SE-specific IgE and IgG were performed by Green Cross LabCell Corporation (Korea). The levels of specific IgE and IgG directed against SA enterotoxin A (SEA) and SA enterotoxin B (SEB) were measured using the immunoCAP system (Pharmacia Diagnostics, Uppsala, Sweden), according to the manufacturer’s instructions. In line with previous publications, SE sensitization was defined as an SEA or SEB value ≥ 0.1 KU/L [6, 9, 10].

### Statistics

Categorical variables were presented as numbers and percentages and were analyzed using Pearson’s chi-square test and Fisher’s exact test. Continuous variables were presented as means ± standard deviations and were analyzed using the Student’s t-test or the Mann–Whitney U test. Bivariate analysis results were plotted on a diagram of variance and evaluated using Pearson’s correlation coefficients. Multivariate analysis was used to determine whether SA carriage and SE IgE-sensitization were correlated with environmental, social, and lifestyle, type 2 inflammation markers and respiratory symptoms by using a logistic regression model. In the Adjust model of risk factors of SA carriage and SE IgE- sensitization, the variables included in the correction were age, sex, body mass index (BMI), smoking status, and three environmental groups. In the adjust model of correlation with type 2 inflammation markers and airway symptoms, the variables included in the correction were age, sex, BMI, smoking status, three environmental groups, asthma, and allergic rhinitis. Results with *P*-values of < 0.05 were considered statistically significant. All analyses were performed using IBM SPSS Statistics (Version 24.0; SPSS Inc., Chicago, IL).

## Results

### General characteristics of the study participants

There were 307 participants in the study (urban, n = 100; suburban, n = 103; rural, n = 104). The baseline characteristics of the participants are presented in Table 1. The mean participant age was 65.12 ± 12.72 (Mean ± standard deviation, SD) years, and 69.1% were female. All participants were examined for SA carriage and SE IgE-sensitization. The overall prevalence of SA carriage was 26.1% (80/307). The overall prevalence of SE-IgE sensitization was 25.7% (79/307) and 20.3% (16/79) were sensitized only to SEA, 39.2% (31/79) were sensitized only to SEB, and 40.5% (32/79) were sensitized to both SEA and SEB. When divided into four groups according to SA carriage and SE IgE-sensitization, it was observed that the SA carriage (+) and SE IgE-sensitization (+) group was 6.5% (20/307), the SA carriage (+) and SE IgE-sensitization (−) group was 19.5% (60/307), and the SA carriage (−) and SE IgE-sensitization (+) was 19.2% (59/307).

**Table 1:** Total participant characteristics

Characteristics	Total, N = 307	%
Baseline characteristics		
Age, year	65.12 ± 12.72	
BMI, kg/m^2^	23.93 ± 3.16	
Sex, female	212/307	69.1
Environment characteristics		
Environmental group		
Urban	100/307	32.6
Sub-urban	104/307	33.9
Rural	103/307	33.6
Home type		
Apartment	72/300	24.0
House	160/300	53.3
Traditional house	68/300	22.7
Year of house construction, year	25.29 ± 16.24	
Proximity to major roadways		
< 50 m	68/298	22.8
≥ 50 m	230/298	77.2
Social and lifestyle characteristics		
Smoking		
Current	30/306	9.8
Ex	40/306	13.1
Never	236/306	77.1
Alcohol drinking		
≥ 1/week	48/266	18.0
< 1/week	218/266	82.0
Exercise		
< 1/week	146/304	48.0
≥ 1/week	158/304	52.0
Dominant diet		
Red meat	55/255	21.6
Fish	31/255	12.2
Vegetable	169/255	66.3

*SA carriage* *Staphylococcus aureus* nasal carriage, *SE* *Staphylococcus aureus* enterotoxin, *SE IgE-sensitization* IgE-sensitization to *Staphylococcus aureus* enterotoxin, *BMI* body mass index. Values are presented as numbers, mean ± SD

### Environmental, social, and lifestyle risk factors of SA carriage and SE IgE-sensitization

The mean age of the participants with SA carriage was 62.53 ± 13.02 years, which indicated that a lower age favored colonization. Environmental factors mainly influenced SA carriage, especially in the urban area group, which had a colonization rate of 38%, a significantly higher rate than in the suburban and rural area groups (suburban: 21.2%, rural: 19.4%). The distance of the participants’ residence from a major road was also identified as a related factor of SA carriage by univariate analysis. (Additional file 1: Table S1). The mean age of the participants with SE IgE-sensitization was 67.78 ± 10.11 years and SE IgE-sensitization group was older than SE IgE-nonsensitization group. SE IgE-sensitization tended to be higher in men (31/95, 32.6%) and in those with a BMI ≥ 25 kg/m^2^ (32/97, 33.0%). However, these findings were not statistically significant (Additional file 1: Table S1).

In multivariate analysis, the environment of the urban area was significantly correlated with SA carriage (odds ratio [OR] 2.309, 95% confidence interval [CI] 1.165–4.575) (Table 2). While, SE IgE-sensitization was increased with age (OR increase/annum, 1.049; 95% CI, 1.021–1.078) and BMI (OR increase/1 kg/m^2^, 1.121; 95% CI, 1.022–1.230). Other than in the urban environmental group, detailed environment, social, and lifestyle factors did not show significant differences in the multivariate analysis (Additional file 1: Table S2).

**Table 2:** Risk factors of SA carriage and SE IgE-sensitization by multivariate analysis

Characteristics	SA carriage		SE IgE-sensitization	
	Adjusted Model* OR (95% CI)	*P* value	Adjusted Model* OR (95% CI)	*P* value
Age (annum)	0.983 (0.962–1.005)	0.123	**1.049 (1.021–1.078)**	**0.001**
BMI (1 kg/m2)	0.955 (0.874–1.044)	0.314	**1.121 (1.022–1.230)**	**0.016**
Sex				
Male	1.483 (0.684–3.216)	0.319	2.093 (0.965–4.549)	0.061
Female	Ref.		Ref.	
Smoking				
Current	0.456 (0.157–1.326)	0.149	1.300 (0.479–3.525)	0.606
Ex	0.593 (0.212–1.657)	0.319	0.458 (0.163–1.284)	0.138
Never	Ref.		Ref.	
Environmental Group				
Urban	**2.309 (1.165–4.575)**	**0.017**	0.960 (0.477–1.932)	0.908
Sub-urban	1.079 (0.519–2.242)	0.838	0.833 (0.408–1.699)	0.614
Rural	Ref.		Ref.	
SA carriage	–		1.087 (0.573–2.061)	0.798
SE IgE-sensitization	1.102 (0.584–2.081)	0.764	–	
SEA IgG (1 mg/L)	**1.025 (1.001–1.049)**	**0.042**	**1.025 (1,000–1.050)**	**0.049**
SEB IgG (1 mg/L)	**1.025 (1.003–1.048)**	**0.026**	**1.027 (1.003–1,051)**	**0.024**

*SA carriage* *Staphylococcus aureus* nasal carriage, *SE* *Staphylococcus aureus* enterotoxin, *SE IgE-sensitization* IgE-sensitization to *Staphylococcus aureus* enterotoxin, *OR* odds ratio, *CI* confidence interval, *BMI* body mass index. Bold indicates significant differences (*P*-value < 0.05). *Adjusted for age, sex, body mass index, smoking status, and environmental group

### Relationship between SA carriage and SE IgE-sensitization and SE IgG

**Fig. 1:**
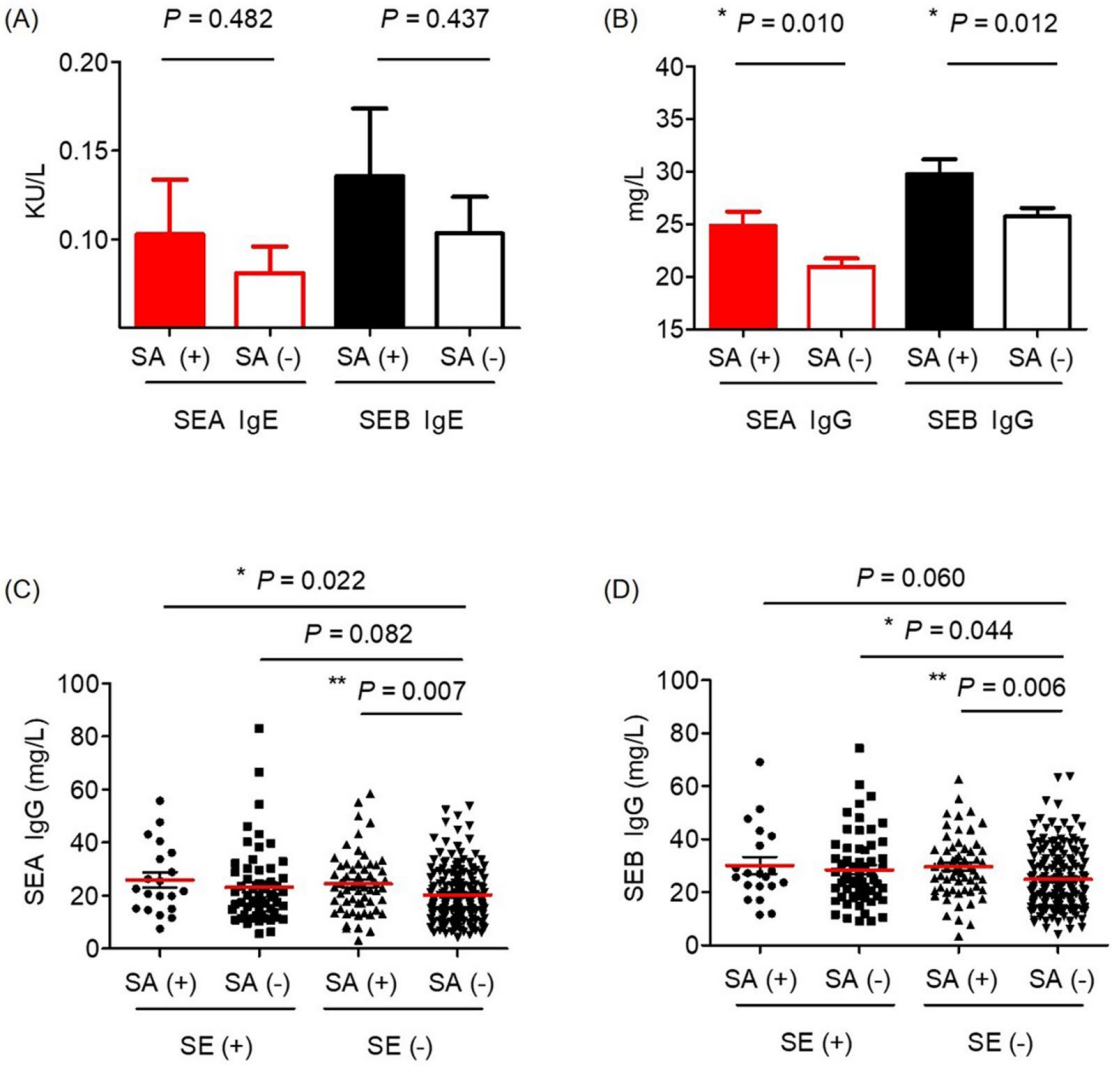
Relationship between *Staphylococcus aureus* nasal carriage, *Staphylococcus aureus* enterotoxin (SE) IgE, and IgG. SA (+) represents the *Staphylococcus aureus* nasal carriage group. **A** *Staphylococcus aureus* enterotoxin A (SEA) IgE and *Staphylococcus aureus* enterotoxin B (SEB) IgE according to SA carriage. **B** SEA IgG and SEB IgG according to SA carriage. **C** SEA IgG according to *Staphylococcus aureus* nasal carriage and SE IgE-sensitization. **D** SEB IgG according to *Staphylococcus aureus* nasal carriage and SE IgE-sensitization. *P < 0.05, **P < 0.01

SA carriage showed a strong correlation with SE IgG levels, but not with SE IgE levels. For SA carriage, SEA IgE (SA carriage vs. SA noncarriage, 0.10 ± 0.28 vs. 0.08 ± 0.23 KU/L; *P* = 0.482) and SEB IgE (0.14 ± 0.34 vs. 0.10 ± 0.31 KU/L, *P* = 0.437) showed no significant increase (Fig. 1A), whereas SEA IgG (24.85 ± 11.83 vs. 20.96 ± 11.43 mg/L; *P* = 0.010) and SEB IgG (29.77 ± 12.46 vs. 25.73 ± 12.22 mg/L; *P* = 0.012) showed a significant increase (Fig. 1B). SA carriage and SE IgE-sensitization were not correlated when multivariate analysis was performed (Table 2).

A strong correlation was observed between SEA IgE and SEB IgE (R = 0.729, *P* < 0.001) and between SEA IgG and SEB IgG (R = 0.809, *P* < 0.001) (Additional file 1: Figure S2A, B). When divided into four groups according to SA carriage and SE IgE-sensitization, other three groups had significantly increased SE IgG compared with the SA carriage (−) and SE IgE-sensitization (−) group (Fig. 1C, D). In multivariate analysis, SE IgG was correlated not only with SA carriage but also with SE IgE-sensitization (Table 2).

### Type 2 immune response according to SA carriage and SE IgE-sensitization

**Fig. 2:**
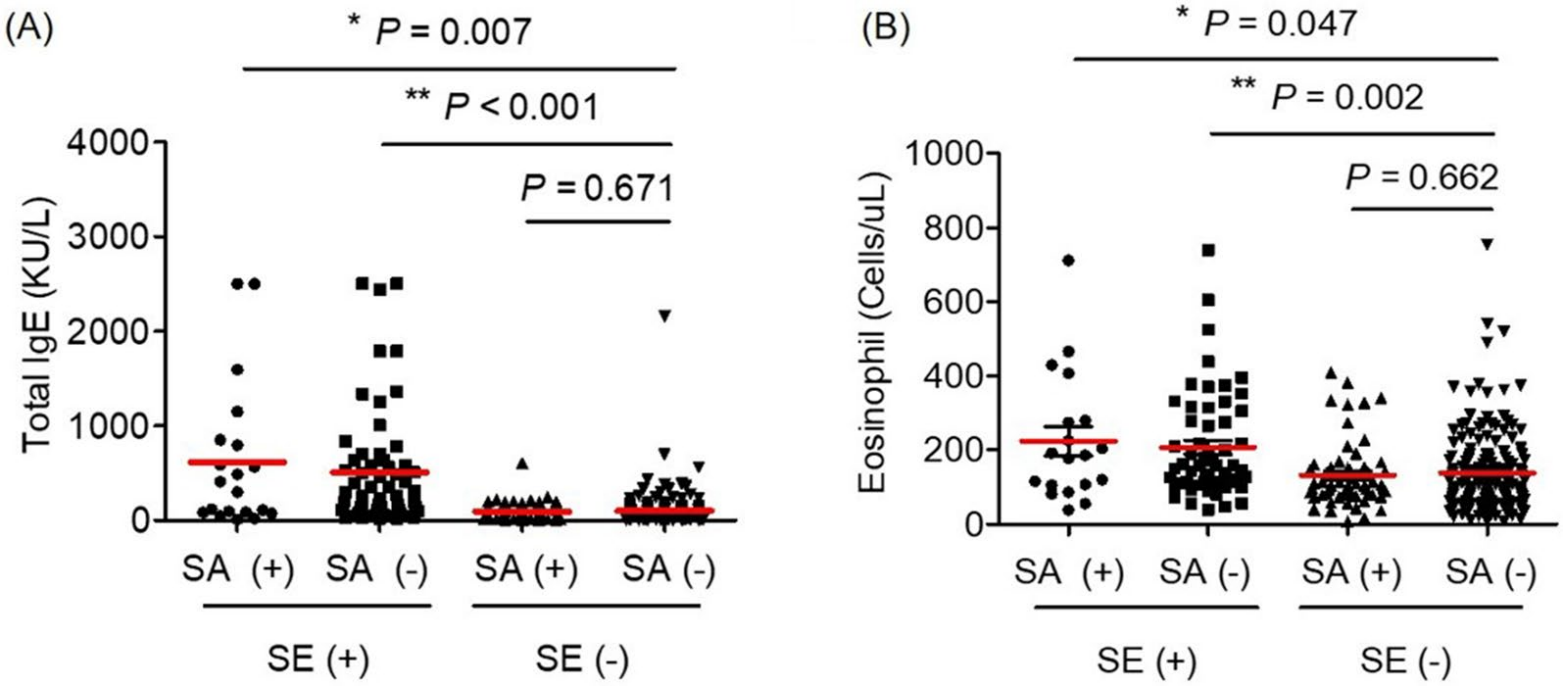
Type 2 inflammation markers according to *Staphylococcus aureus* nasal carriage and SE IgE-sensitization. SE (+) represents the SE IgE-sensitization group, and SA (+) represents the *Staphylococcus aureus* nasal carriage group. **A** Total IgE, **B** Blood eosinophils, *P < 0.05, **P < 0.01

In the SE IgE-sensitization group, total IgE (SE IgE-sensitization vs. SE IgE-nonsensitization, 536.88 ± 659.79 vs. 100.25 ± 170.27 KU/L, *P* < 0.001), blood eosinophil count (212.11 ± 150.31 vs. 137.12 ± 105.49 cells/µL, *P* < 0.001), and blood basophil count (37.36 ± 23.93 vs. 30.25 ± 20.93 cells/µL, *P* = 0.015) were all significantly elevated compared with the SE IgE-nonsensitization group (Fig. 2, Additional file 1: Table S3). This significant correlation between SE IgE-sensitization and type 2 inflammation was still pronounced when variables were controlled through multivariate analysis (Table 3). However, total IgE and blood eosinophil count were not dependent on the SA carriage group both in the univariate analysis (Fig. 2, Additional file 1: Table S3) and multivariate analysis (Table 3).

**Table 3:** Association with type 2 inflammation markers and *Staphylococcus aureus* groups by multivariate analysis

Characteristics	SA carriage		SE IgE-sensitization	
	Adjusted model* OR (95% CI)	*P* value	Adjusted model* OR (95% CI)	*P* value
Total IgE, KU/L				
≥ 200	1.133 (0.551–2.330)	0.735	**11.690 (5.535–24.686)**	**< 0.001**
< 200, ≥ 100	1.328 (0.681–2.589)	0.406	**2.415 (1.098–5.312)**	**0.028**
< 100	Ref.		Ref.	
Blood eosinophil, × 10^9^/L				
≥ 300	1.247 (0.517–3.007)	0.623	**4.371 (1.817–10.516)**	**0.001**
< 300, ≥ 150	0.822 (0.420–1.609)	0.567	1.550 (0.787–3.054)	0.205
< 150	Ref.		Ref.	

*SA carriage* *Staphylococcus aureus* nasal carriage, *SE* *Staphylococcus aureus* enterotoxin, *SE IgE-sensitization* IgE-sensitization to *Staphylococcus aureus* enterotoxin, *OR* odds ratio, *CI* confidence interval. Bold indicates significant differences (*P*-value < 0.05). *Adjusted for age, sex, body mass index, smoking status, environmental group, asthma and allergic rhinitis

### Respiratory symptoms according to SA carriage and SE IgE-sensitization

In this study, although eosinophil count and total IgE were increased in the SE IgE-sensitization group, neither SA carriage nor SE IgE-sensitization was causally related to asthma, allergic rhinitis, dyspnea within the last 12 months and wheezing within the last 12 months (Additional file 1: Table S3). The multivariate analysis showed that cough was associated with allergic rhinitis. Furthermore, smoking was the most important association factor for sputum production, along with allergic rhinitis, aging and obesity as observed by multivariate analysis (Additional file 1: Table S4).

The SA carriage group was independently associated with cough (OR, 3.044; 95% CI, 1.137–8.153; *P* = 0.027) and sputum production (OR, 2.429; 95% CI, 1.008–5.854; *P* = 0.048) whereas the SE IgE-sensitization group was independently associated with sputum production (OR, 2.452; 95% CI, 1.066–5.640; *P* = 0.035) (Table 4).

**Table 4:** Association with respiratory symptoms and *Staphylococcus aureus* groups by multivariate analysis

	Cough		Sputum production	
	Adjusted Model* OR (95% CI)	*P* value	Adjusted model* OR (95% CI)	*P* value
SA carriage	**3.044 (1.137–8.153)**	**0.027**	**2.429 (1.008–5.854)**	**0.048**
SE IgE-sensitization	1.943 (0.716–5.273)	0.192	**2.452 (1.066–5.640)**	**0.035**
SA carriage and SE IgE-sensitization group				
SA carriage (+), SE IgE-sensitization (+)	**6.303 (1.231–32.272)**	**0.027**	**7.258 (2.163–24.361)**	**0.001**
SA carriage (+), SE IgE-sensitization (−)	**3.824 (1.093–13.383)**	**0.036**	1.350 (0.398–4.574)	0.630
SA carriage (−), SE IgE-sensitization (+)	2.632 (0.693–9.990)	0.155	1.586 (0.560–4.493)	0.386
SA carriage (−), SE IgE-sensitization (−)	Ref.		Ref.	

*SA carriage* *Staphylococcus aureus* nasal carriage, *SE* *Staphylococcus aureus* enterotoxin, *SE IgE-sensitization* IgE-sensitization to *Staphylococcus aureus* enterotoxin, *OR* odds ratio, *CI* confidence interval. Bold indicates significant differences (*P*-value < 0.05). *Adjusted for age, sex, body mass index, smoking status, environmental group, asthma and allergic rhinitis

The associations of cough and sputum production were investigated by dividing the participants into four groups according to the indicators of SA carriage and SE IgE-sensitization. After adjusting for other risk factors, the SA carriage (+) and SE IgE-sensitization (+) group (OR, 6.303; 95% CI, 1.231–32.272; *P* = 0.027) and the SA carriage (+) and SE IgE-sensitization (−) group (OR, 3.824; 95% CI, 1.093–13.383; *P* = 0.036) were identified as association factors for cough (Table 4). In contrast, the only association factor that was found for sputum production was the SA carriage (+) and SE IgE-sensitization (+) group (OR, 7.258; 95% CI, 2.163–24.361; *P* = 0.001) (Table 4). When the cough was divided depending on the presence or absence of sputum production, the SE IgE-sensitization group showed an increased risk of cough with sputum production (OR, 5.638; 95% CI, 1.314–24.198, *P* = 0.020) and the SA carriage group showed an increased risk of cough without sputum production (OR, 5.257; 95% CI, 1.668–16.565, *P* = 0.005) (Additional file 1: Table S5).

## Discussion

In our study, we evaluated the differences between SA carriage and SE IgE-sensitization in the Korean adult population. Specifically, we found that: (1) SA carriage was associated with the urban environment, and SE IgE-sensitization was associated with host factors, such as age and BMI. (2) There was no significant correlation between SA carriage and SE IgE-sensitization. (3) SA carriage had different effects depending on whether SE IgE-sensitization was present or not. SA carriage in SE IgE-sensitization induced type 2 inflammation and increased sputum and cough, whereas SA carriage in SE IgE non-sensitization increased dry cough symptoms without inducing type 2 inflammation.

In this community-based study, urban environmental factors had a more significant effect on SA carriage than individual host factors. In a study based on the National Health and Nutrition Examination Survey (NHANES), 2001–2004 data showed that mixtures within metals, phthalates, and phenols were associated with SA carriage [11]. Particulate matter (PM), such as black carbon, has also been reported to be associated with the nasal microbiome change including SA in the nasopharynx [12]. Other studies indicated that SA achieved higher levels of nasal colonization in corporate settings or hospital environments, suggesting that human contact is a crucial transmission method [13, 14]. The urban area in this study represented high population density and high concentration of PM, as well as a high concentration of metal substances and VOCs from the industrial complex as it was adjacent to the Pohang Industrial Complex. The high prevalence of SA carriage in the urban group was likely due to a combination of the above factors. Although The Pohang city has the specificity of Pohang Industrial Complex, in most metropolitan cities, high air pollution level and high population density problems exist together. So we think the results of our study are applicable to general large cities. The difference according to the types of major pollutants by city will need to be confirmed through additional research. Other factors that have previously been reported to affect the SA carriage include underlying diseases, such as immune deficiency, autoimmune disease and diabetes with dialysis [15], and seasonal factors, such as temperature and humidity [16]. However, this study was conducted in the general population, and the prevalence of such underlying diseases was low. And even though hypertension and diabetes had a relatively high prevalence, they did not show any association with SA carriage and SE IgE-sensitization. In addition, this study was carried out in all participants at relatively similar temperature, humidity, and seasonal conditions through cross-sectional studies in similar regions.

Interestingly, there was no significant correlation between SA carriage and SE IgE-sensitization. This result can be attributed to two aspects. First aspects, SA carriage do not induce SE IgE-sensitization in everyone and do not induce type 2 inflammation in everyone. Although SE IgG was increased in the SA carriage, there was no significant increase in SE IgE and type 2 inflammation markers in the SA carriage group. Who leads to SE IgE-sensitization in the SA carriage group is important for understanding airway eosinophilic inflammation, one of the important phenotypes of airway inflammation. The environment group was not a risk factor for SE IgE-sensitization, and host factors such as age and obesity were the significant risk factors. When limited to SA carriage group, SE IgE-sensitization was significant higher in older ages and male (data not shown). These results were similar to the findings of an epidemiological study of community-based adult populations in Korea, suggesting males, smoking, and old age were major risk factors of SE IgE-sensitization [17]. Although smoking was considered the most important risk factor for SE IgE-sensitization [17, 18], the proportion of smokers in this study was lower than in previous studies. It is necessary to study further why the SA carriage does not directly correlate with SE IgE, but the risk of sensitization varies depending on the host factor. It may provide clues to the mechanism of airway eosinophilic inflammation in patients without atopy.

Second aspects, there was also a SE IgE-sensitization group that was not accompanied by SA carriage. Various routes of SE IgE-sensitization could be the cause of this difference. Considering the increase in SE IgG in the SE IgE-sensitization group, it is possible that the SE IgE-sensitization without SA carriage group had been sensitized to SE in areas other than the airway. Skin is known as an important sensitization route for allergens [19], and skin is a common place of colonization in SA [15]. Moreover, the relationship between atopic dermatitis and SA colonization in the skin seems to show a much stronger correlation than airway [20].

In this study, SA carriage showed different effects depending on the presence or absence of SE IgE sensitization. In the SE IgE-sensitized group, even though the SA carriage was not accompanied, total IgE and blood eosinophil count were elevated. But the respiratory symptoms, especially sputum production, were correlated when the SA carriage was accompanied. On the other hand, the SA carriage without SE IgE-sensitization group was also associated with respiratory symptoms but showed a different pattern from the SE IgE-sensitization group. SA carriage without SE IgE-sensitization increased the prevalence of cough more than three weeks regardless of type 2 inflammation. This association was still significant even with the exception of smoking, asthma, and allergic rhinitis, which are common causes of cough [21]. Urban area may have a high prevalence of cough for reasons other than SA carriage, such as air pollution and infection transmission due to high population density. However, in our study, SA carriage showed a stronger correlation with cough than environmental group, and it was still found as an independent risk factor through multivariate analysis. Therefore, SA carriage may be one of the important factors explaining the high prevalence of cough in urban areas. SA proteins are known to primarily induce type 1 and type 3 immune responses characterized by the release of IFN-γ and IL-17 along with the production of specific IgGs [22–24]. SA may also have secondary effects, which interacts with other components of the nasal microbiome and induces changes in the intranasal environment [1, 25]. Further research is needed to determine whether cough development is a direct result of SA or a secondary effect.

With regard to the characteristics of the study participants, approximately 70% were over 60 year-old, and this needs to be considered when comparing our findings with those of previous studies on adolescents or middle-aged adults [6, 7, 26]. Furthermore, in contrast to other studies, there were fewer participants who smoked or were obese in our study. The prevalence of asthma and upper airway disease were generally similar to the prevalence of asthma, and this was evaluated based on “ever diagnosis by doctor” in the general Korean population [27, 28]. In this study, 7.2% of participants were classified as having a cough for more than three weeks, which included subacute cough and chronic cough. This was a higher percentage than was previously reported for chronic cough in a Korean study (2.6–4.6%) [29].

Our study did not show the previously established relationships between SA and airway diseases, including asthma and allergic rhinitis. Although the relationship between chronic airway disease and SA is well known, it can often be found that the relationship between SA carriage and chronic airway disease is not significant in general population studies, especially among adults [6, 7]. The reason that asthma and allergic rhinitis in our study were not associated with SA carriage and SE IgE-sensitization may be due to the fact that patients with asthma or allergic rhinitis in this study were mainly mild, asymptomatic and mixed with a variable phenotypes.

Strengths of the study include that SA carriage and SE IgE-sensitization were confirmed in a relatively large number of participants, and the analysis was performed by considering various environmental factors, including pollution levels and population densities. Thus, our results focused on the environmental factors, which have not been well-studied. In addition, unlike previous studies, we divided the SA carriage and SE IgE-sensitization into the effect of SA. So, the difference in the impact of SA carriage and SE IgE-sensitization on type 2 inflammation and respiratory symptoms could be investigated.

Nevertheless, this study also has limitations. First, due to the cross-sectional nature of the study, the causal relationship was not accessible. In addition, it was not possible to determine whether colonization by SA was temporary or permanent. Second, the survey was conducted using a questionnaire, and therefore, it was not possible to decide on the phenotypes of asthma or allergic rhinitis. Finally, our study measured only sensitization to SEA and SEB; among 23 components of SEs, SE IgE-sensitization may have been underestimated. However, only SEA and SEB measurements can representatively show trends in risk factors and health effects [30].

## Conclusion

SA carriage was found to be dependent on environmental factors and SE IgE-sensitization on individual host characteristics. There was no significant correlation between SA carriage and SE IgE-sensitization. The effect of SA carriage on the respiratory tract is not the same and causes different patterns of airway inflammation and symptoms depending on whether or not SE is sensitized. In addition, SA carriage and SE IgE-sensitization showed a synergistic effect on the prevalence of cough and sputum. So, when interpreting the means of SA carriage and SE IgE as biomarkers for type 2 inflammation or chronic airway diseases, it is suggested to interpret them differently.

## Supplementary Information

**Additional file 1.** **Table S1.** Risk factors of SA carriage and SE IgE-sensitization by univariate analysis. ** Table S2.** Details environmental, social and lifestyle risk factors of SA carriage and SE IgE-sensitization by multivariate analysis. **Table S3.** Respiratory symptoms, underlying disease, and laboratory findings according to SA carriage and SE IgE-sensitization. **Table S4.** Environment, social and lifestyle, and comorbidity association factors of persistent cough and sputum production by multivariate analysis. **Table S5.** Association with persistent cough and Staphylococcus aureus 4 groups by multivariate analysis. **Figure S1.** Three population groups according to environmental exposure. **Figure S2.** Correlation between Staphylococcus aureus enterotoxin (SE) IgE and IgG according to Staphylococcus aureus nasal carriage.

## Data Availability

The data that support the findings of this study are available on request from the corresponding author, upon reasonable request.

## Abbreviations

SA: *Staphylococcus aureus*
SA carriage: *Staphylococcus aureus* Nasal carriage
SE: *Staphylococcus aureus* Enterotoxin
SE IgE-sensitization: IgE sensitization to *Staphylococcus aureus* enterotoxin
SEA: *Staphylococcus aureus* Enterotoxin A
SEB: *Staphylococcus aureus* Enterotoxin B
VOC: Volatile organic compounds
BMI: Body mass index
PM: Particulate matter
