# Supplementary material for: Differences between Staphylococcus aureus nasal carriage and IgE-sensitization to Staphylococcus aureus enterotoxin on risk factors and effects in adult population
Source: Allergy Asthma Clin Immunol. 2022 Jan 31;18:6. doi: 10.1186/s13223-022-00648-4 (PMC8805341; doi:10.1186/s13223-022-00648-4)
Supplement: Supplementary file 1 — Additional file 1. Table S1. Risk factors of SA carriage and SE IgE-sensitization by univariate analysis. Table S2. Details environmental, social and lifestyle risk factors of SA carriage and SE IgE-sensitization by multivariate analysis. Table S3. Respiratory symptoms, underlying disease, and laboratory findings according to SA carriage and SE IgE-sensitization. Table S4. Environment, social and lifestyle, and comorbidity association factors of persistent cough and sputum production by multivariate analysis. Table S5. Association with persistent cough and Staphylococcus aureus 4 groups by multivariate analysis. Figure S1. Three population groups according to environmental exposure. Figure S2. Correlation between Staphylococcus aureus enterotoxin (SE) IgE and IgG according to Staphylococcus aureus nasal carriage. [file 13223_2022_648_MOESM1_ESM.docx]

**Table S1. Risk factors of SA carriage and SE IgE-sensitization by univariate analysis**

|  | SA carriage | | | SE IgE-sensitization | | |
| --- | --- | --- | --- | --- | --- | --- |
|  | SA carriage  N=80 (26.1) | SA noncarriage  N=227 (73.9) | *P* value | SE IgE-sensitization N=79 (25.7) | SE IgE non-sensitization N=228 (74.3) | *P* value |
| **Baseline characteristics** |  |  |  |  |  |  |
| Age, year | **62.53 ± 13.02** | **66.53 ± 12.52** | **0.034** | **67.78 ± 10.11** | **64.20 ± 13.41** | **0.014** |
| BMI, kg/m^2^ | 23.81 ± 3.10 | 23.97 ± 3.18 | 0.695 | 24.46 ± 3.36 | 23.74 ± 3.07 | 0.093 |
| Sex, female | 55/212 (25.9) | 157/212 (74.1) | 0.945 | 48/212 (22.6) | 164/212 (77.4) | 0.064 |
| **Environment characteristics** |  |  |  |  |  |  |
| Environmental Group |  |  | **0.004** |  |  | 0.597 |
| Urban | 38/100 (38.0) | 62/100 (62.0) |  | 25/100 (25.0) | 75/100 (75.0) |  |
| Sub-urban | 22/104 (21.2) | 82/104 (78.8) |  | 23/104 (23.1) | 80/104 (76.9) |  |
| Rural | 20/103 (19.4) | 83/103 (80.6) |  | 30/103 (29.1) | 73/103 (70.9) |  |
| Home type |  |  | 0.818 |  |  | 0.102 |
| Apartment | 21/72 (29.2) | 51/72 (70.8) |  | 14/72 (19.4) | 58/72 (80.6) |  |
| House | 41/160 (25.6) | 119/160 (74.4) |  | 48/160 (30.0) | 112/160 (70.0) |  |
| Traditional house | 17/68 (25.0) | 51/68 (75.0) |  | 13/68 (19.1) | 55/68 (80.9) |  |
| Year of house construction, year | 25.28 ± 12.93 | 25.30 ± 17.30 | 0.991 | 23.68 ± 19.02 | 25.83 ± 15.23 | 0.332 |
| Proximity to major roadways |  |  | **0.024** |  |  | 0.452 |
| <50 m | 25/68 (36.8) | 43/68 (63.2) |  | 19/68 (27.9) | 49/68 (72.1) |  |
| ≥50 m | 53/230 (23.0) | 177/230 (80.5) |  | 54/230 (23.5) | 176/230 (76.5) |  |
| **Social and lifestyle characteristics** |  |  |  |  |  |  |
| Smoking |  |  | 0.773 |  |  | 0.583 |
| Current | 7/30 (23.3) | 21/30 (76.7) |  | 10/30 (33.3) | 20/30 (66.7) |  |
| Ex | 9/40 (22.5) | 31/40 (77.5) |  | 10/40 (25.0) | 30/40 (75.0) |  |
| Never | 64/236 (27.1) | 172/236 (72.9) |  | 58/236 (24.6) | 178/236 (75.4) |  |
| Alcohol Drinking |  |  | 0.391 |  |  | 0.084 |
| ≥1/week | 15/48 (31.3) | 33/48 (68.8) |  | 17/48 (35.4) | 31/48 (64.6) |  |
| <1/week | 55/218 (25.2) | 163/218 (74.8) |  | 51/218 (23.4) | 167/218 (76.6) |  |
| Exercise |  |  | 0.913 |  |  | 0.431 |
| <1/week | 38/146 (26.0) | 108/146 (74.0) |  | 34/146 (23.3) | 112/146 (76.7) |  |
| ≥1/week | 42/158 (26.6) | 116/158 (73.4) |  | 43/158 (27.2) | 115/158 (72.8) |  |
| Dominant Diet |  |  | **0.033** |  |  | 0.582 |
| Red meat | 21/55 (38.2) | 34/55 (61.8) |  | 13/55 (23.6) | 42/55 (76.4) |  |
| Fish | 11/31 (35.5) | 20/31 (64.5) |  | 10/31 (32.3) | 21/31 (67.7) |  |
| Vegetable | 37/169 (21.9) | 132/169 (78.1) |  | 40/169 (23.7) | 129/169 (76.3) |  |

*Abbreviations:* SA carriage: staphylococcus aureus nasal carriage; SE: staphylococcus aureus enterotoxin; SE IgE-sensitization: IgE-sensitization to staphylococcus aureus enterotoxin; BMI: body mass index. Values are presented as numbers, mean ± SD. Bracket are presented as positive rate of SA carriage or SE IgE-sensitization. Bold indicates significant differences (*P*-value < 0.05).

**Table S2. Details environmental, social and lifestyle risk factors of SA carriage and SE IgE-sensitization by multivariate analysis**

| Characteristics | SA carriage | | SE sensitization | |
| --- | --- | --- | --- | --- |
|  | Adjusted Model*  OR (95% CI) | *P* value | Adjusted Model*  OR (95% CI) | *P* value |
| **Home type** |  |  |  |  |
| Apartment | 0.537 (0.215 – 1.342) | 0.183 | 1.309 (0.476 – 3.599) | 0.601 |
| House | 0.796 (0.383 – 1.651) | 0.540 | 1.637 (0.757 – 3.541) | 0.210 |
| Traditional house | Ref |  | Ref |  |
| **Proximity to major roadways** |  |  |  |  |
| < 50 m | 1.595 (0.866 – 2.936) | 0.134 | 1.292 (0.657 – 2.541) | 0.458 |
| ≥ 50 m | Ref |  | Ref |  |
| **Alcohol Drinking** |  |  |  |  |
| ≥ 1/week | 1.459 (0.652 – 3.263) | 0.358 | 1.898 (0.829 – 4.345) | 0.129 |
| < 1/week | Ref |  | Ref |  |
| **Exercise** |  |  |  |  |
| < 1/week | 1.009 (0.584 – 1.743) | 0.975 | 0.794 (0.445 – 1.418) | 0.436 |
| ≥ 1/week | Ref |  | Ref |  |
| **Dominant Diet** |  |  |  |  |
| Red meat | 2.036 (0.933 – 4,442) | 0.074 | 1.104 (0.465 – 2.623) | 0.822 |
| Fish | 1.960 (0.794 – 4.837) | 0.144 | 1.425 (0.560 – 3.623) | 0.457 |
| Vegetable | Ref |  | Ref |  |

*Abbreviations:* SA carriage: staphylococcus aureus nasal carriage; SE: staphylococcus aureus enterotoxin; SE IgE-sensitization: IgE-sensitization to staphylococcus aureus enterotoxin; OR: odd ratio; CI: confidence interval. Bold indicates significant differences (*P*-value < 0.05). *The adjusted model was adjusted for age, sex, body mass index, smoking status, and environmental group.

**Table S3. Respiratory symptoms, underlying disease, and laboratory findings according to SA carriage and SE IgE-sensitization**

|  | SA carriage | | | SE IgE-sensitization | | | |
| --- | --- | --- | --- | --- | --- | --- | --- |
|  | SA carriage  N=80 (26.1) | SA noncarriage  N=227 (73.9) | *P* value | SE IgE-sensitization N=79 (25.7) | SE IgE non-sensitization N=228 (74.3) | | *P* value |
| **Respiratory symptoms** |  |  |  |  |  | |  |
| Cough |  |  | **0.048** |  |  | | **0.018** |
| (+) | 10/22 (45.5) | 12/22 (54.5) |  | 10/22 (45.5) | 12/22 (54.5) | |  |
| (-) | 67/259 (25.9) | 192/259 (74.1) |  | 59/259 (22.8) | 200/259 (77.2) | |  |
| Sputum production |  |  | 0.172 |  |  | | **0.004** |
| (+) | 13/36 (36.1) | 23/36 (63.9) |  | 16/36 (44.4) | 20/36 (55.6) | |  |
| (-) | 67/264 (25.4) | 197/264 (74.6) |  | 59/264 (22.3) | 205/264 (77.7) | |  |
| Dyspnea |  |  | 0.880 |  |  | | 0.657 |
| (+) | 12/47 (25.5) | 35/47 (74.5) |  | 13/47 (27.7) | 34/47 (72.3) | |  |
| (-) | 67/252 (26.6) | 185/252 (73.4) |  | 62/252 (24.6) | 190/252 (75.4) | |  |
| Wheezing |  |  | 0.086 |  |  | | 0.952 |
| (+) | 2/25 (12.0) | 22/25 (88.0) |  | 6/25 (24.0) | 19/25 (76.0) | |  |
| (-) | 76/273 (27.8) | 197/273 (72.2) |  | 67/273 (24.5) | 206/273 (75.5) | |  |
| **Underlying diseases** |  |  |  |  |  | |  |
| asthma |  |  | 0.412 |  |  | | 0.445 |
| (+) | 3/17 (17.6) | 14/17 (82.4) |  | 3/17 (17.6) | 14/17 (82.4) | |  |
| (-) | 77/289 (26.6) | 212/289 (73.4) |  | 75/289 (26.0) | 214/289 (74.0) | |  |
| allergic rhinitis |  |  | 0.360 |  |  | | 0.588 |
| (+) | 12/37 (32.4) | 25/37 (67.6) |  | 8/37 (21.6) | 29/37 (78.4) | |  |
| (-) | 68/268 (25.4) | 200/268 (74.6) |  | 69/268 (25.7) | 199/268 (74.3) | |  |
| HTN |  |  | 0.414 |  | |  | 0.235 |
| (+) | 26/112 (23.2) | 86/112 (76.8) |  | 33/112 (29.5) | 79/112 (70.5) | |  |
| (-) | 53/193 (27.5) | 140/193 (72.5) |  | 45/193 (23.3) | 148/193 (76.7) | |  |
| Diabetes |  |  | 0.763 |  |  | | 0.600 |
| (+) | 12/49 (24.5) | 37/49 (75.5) |  | 14/49 (28.6) | 35/49 (71.4) | |  |
| (-) | 68/256 (26.6) | 188/256 (73.4) |  | 64/256 (25.0) | 192/256 (75.0) | |  |
| **Laboratory findings** |  |  |  |  |  | |  |
| SEA IgE, KU/L | 0.10 ± 0.28 | 0.08 ± 0.23 | 0.482 | **0.28 ± 0.42** | **0.02 ± 0.02** | | **<0.001** |
| SEB IgE, KU/L | 0.14 ± 0.34 | 0.10 ± 0.31 | 0.437 | **0.38 ± 0.55** | **0.02 ± 0.02** | | **<0.001** |
| SEA IgG, mg/L | **24.85 ± 11.83** | **20.96 ± 11.43** | **0.010** | 23.88 ± 13.94 | 21.31 ± 10.69 | | 0.091 |
| SEB IgG, mg/L | **29.77 ± 12.46** | **25.73 ± 12.22** | **0.012** | 28.89 ± 13.74 | 26.05 ± 11.84 | | 0.079 |
| Total WBC count, x 10^9^/L | 5.59 ± 1.61 | 5.73 ± 1.70 | 0.511 | **6.09 ± 1.63** | **5.56 ± 1.67** | | **0.017** |
| Hemoglobin, g/dL | 13.43 ± 1.47 | 13.29 ± 1.42 | 0.471 | 13.34 ± 1.53 | 13.32 ± 1.40 | | 0.914 |
| Platelet, x 10^9^/L | 228.24 ±89.22 | 232.75 ± 67.58 | 0.639 | 236.78 ± 93.16 | 229.77 ± 65.79 | | 0.467 |
| Neutrophil count, x 10^9^/L | 3.04 ± 1.21 | 3.13 ± 1.32 | 0.583 | 3.30 ± 1.31 | 3.04 ± 1.28 | | 0.135 |
| Lymphocyte count, x 10^9^/L | 2.08 ± 0.63 | 2.10 ± 6.26 | 0.761 | 2.19 ± 0.57 | 2.06 ± 0.64 | | 0.129 |
| Eosinophil count, x 10^9^/L | 0.15 ± 0.12 | 0.16 ± 0.13 | 0.909 | **0.21 ± 0.15** | **0.14 ± 0.11** | | **<0.001** |
| Basophil count, x 10^9^/L | 0.032 ± 0.020 | 0.032 ± 0.022 | 0.864 | **0.037 ± 0.024** | **0.030 ± 0.021** | | **0.015** |
| Total IgE, KU/L | 224.11 ± 450.61 | 208.55 ± 397.27 | 0.772 | **536.88 ± 659.79** | **100.45 ± 170.27** | | **<0.001** |
| BUN, mg/dL | 15.88 ± 4.66 | 15.76 ± 4.89 | 0.850 | 15.83 ± 4.53 | 15.78 ± 4.94 | | 0.937 |
| Creatinine, mg/dL | 0.76 ± 0.18 | 0.81 ± 0.26 | 0.107 | 0.81 ± 0.19 | 0.79 ± 0.25 | | 0.639 |

*Abbreviations:* SA carriage: staphylococcus aureus nasal carriage; SE: staphylococcus aureus enterotoxin; SE IgE-sensitization: IgE-sensitization to staphylococcus aureus enterotoxin. Values are presented as numbers, mean ± SD. Bracket are presented as positive rate of SA carriage or SE IgE-sensitization. Bold indicates significant differences (*P*-value < 0.05).

**Table S4. Environment, social and lifestyle, and comorbidity association factors of persistent cough and sputum production by multivariate analysis**

| Characteristics | Persistent Cough | | Sputum production | |
| --- | --- | --- | --- | --- |
|  | OR (95% CI) | *P* value | OR (95% CI) | *P* value |
| **Age (annum)** | 1.024 (0.983 – 1.068) | 0.255 | **1.063 (1.022 – 1.104)** | **0.002** |
| **BMI (1kg/m2)** | 1.024 (0.878 – 1.194) | 0.764 | **1.166 (1.023 – 1.329)** | **0.022** |
| **Sex** |  |  |  |  |
| Male | 1.310 (0.341 – 5.029) | 0.694 | 0.798 (0.241- 2.636) | 0.711 |
| Female | Ref |  | Ref |  |
| **Smoking** |  |  |  |  |
| Current | 1.226 (0.241 – 6.231) | 0.806 | **12.227 (3.436 – 43.517)** | **<0.001** |
| Ex | 0.234 (0.022 – 2.470) | 0.227 | 1.338 (0.284 – 6.306) | 0.713 |
| Never | Ref |  | Ref |  |
| **Environmental Group** |  |  |  |  |
| Urban | 0.957 (0.289 – 3.176) | 0.943 | 1.356 (0.462 – 3.977) | 0.579 |
| Sub-Urban | 0.990 (0.299 – 3.271) | 0.986 | 1.954 (0.698 – 5.469) | 0.202 |
| Rural | Ref |  | Ref |  |
| **Asthma** | 1.248 (0.223 – 6.997) | 0.801 | 3.285 (0.844 – 12.782) | 0.086 |
| **Allergic rhinitis** | **3.583 (1.159 – 11.078)** | **0.027** | **3.224 (1.158 – 8.975)** | **0.025** |

*Abbreviations:* OR: odds ratio; CI: confidence interval; BMI: Body mass index. Bold indicates significant differences (*P*-value < 0.05). *The adjusted model was adjusted for age, sex, body mass index, smoking status, environmental group, asthma and allergic rhinitis.

**Table S5. Association with persistent cough and Staphylococcus aureus 4 groups by multivariate analysis**

|  | Cough with sputum | | Cough without sputum | |
| --- | --- | --- | --- | --- |
|  | Adjusted Model*  OR (95% CI) | *P* value | Adjusted Model*  OR (95% CI) | *P* value |
| **SA carriage** | 1.655 (0.398 – 6.885) | 0.488 | **5.257 (1.668 – 16.565)** | **0.005** |
| **SE IgE-sensitization** | **5.638 (1.314 – 24.198)** | **0.020** | 0.660 (1.147 – 2.954) | 0.587 |
| **SA carriage and SE sensitization group** |  |  |  |  |
| SA carriage (+), SE IgE-sensitization (+) | **11.719 (1.295 – 106.042)** | **0.029** | 2.572 (0.273 – 24.202) | 0.409 |
| SA carriage (+), SE IgE-sensitization (-) | 1.978 (0.239 – 16.382) | 0.527 | **6.170 (1.718 – 22.159)** | **0.005** |
| SA carriage (-), SE IgE-sensitization (+) | 6.137 (0.896 – 42.012) | 0.065 | 0.751 (0.111 – 5.070) | 0.768 |
| SA carriage (-), SE IgE-sensitization (-) | Ref |  | Ref |  |

*Abbreviations:* SA carriage: staphylococcus aureus nasal carriage; SE: staphylococcus aureus enterotoxin; SE IgE-sensitization: IgE-sensitization to staphylococcus aureus enterotoxin; OR: odds ratio; CI: confidence interval. Bold indicates significant differences (*P*-value < 0.05). *Adjusted for age, sex, body mass index, smoking status, environmental group, asthma and allergic rhinitis.


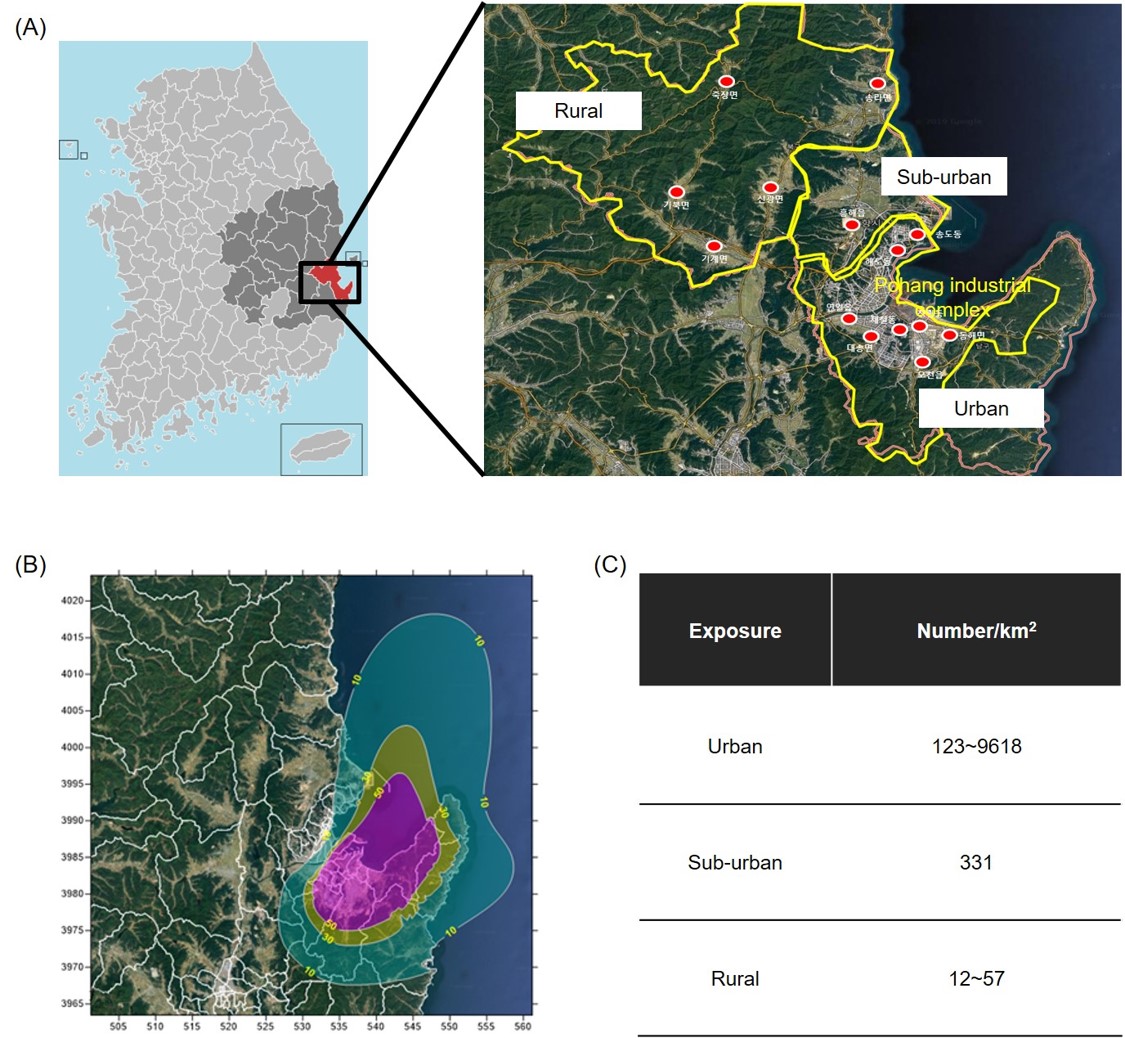


**Figure S1. Three population groups according to environmental exposure.** (A) Three environmental groups and their distance from Pohang Industrial Complex, (B) Atmospheric diffusion modeling by annual average PM10 (µg/m3). (C) Population density according to the environmental group (persons/km2).


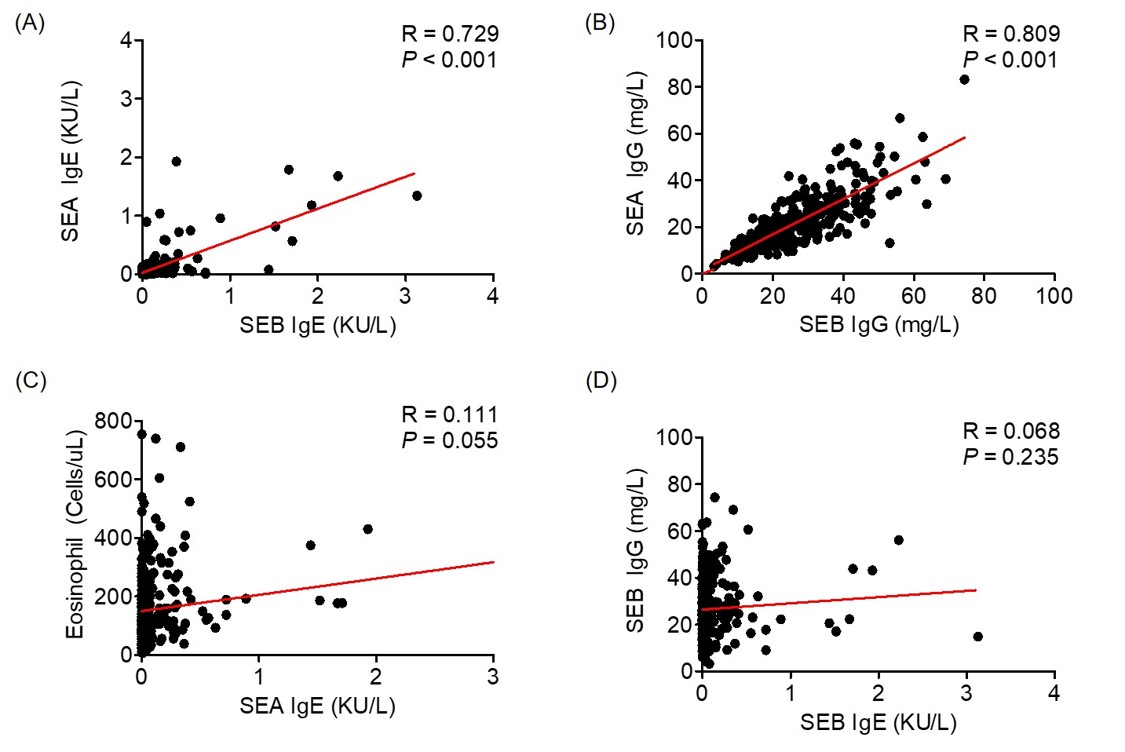


**Figure S2. Correlation between *Staphylococcus aureus* enterotoxin (SE) IgE and IgG according to *Staphylococcus aureus* nasal carriage.** (A) Correlation with *Staphylococcus aureus* enterotoxin A (SEA) IgE and *Staphylococcus aureus* enterotoxin B (SEB) IgE. (B) Correlation with SEA IgG and SEB IgG, (C) Correlation with SEA IgE and SEA IgG. (D) Correlation with SEB IgE and SEB IgG.
